# Immunotherapeutic target expression on breast tumors can be amplified by hormone receptor antagonism: a novel strategy for enhancing efficacy of targeted immunotherapy

**DOI:** 10.18632/oncotarget.15812

**Published:** 2017-03-01

**Authors:** Ritika Jaini, Matthew G. Loya, Charis Eng

**Affiliations:** ^1^ Genomic Medicine Institute, Lerner Research Institute, Cleveland, OH 44195, USA; ^2^ Taussig Cancer Institute, Cleveland Clinic, Cleveland, OH 44195, USA; ^3^ Cleveland Clinic Lerner College of Medicine, Cleveland Clinic, Cleveland, OH 44195, USA; ^4^ Department of Genetics and Genome Sciences, Case Western Reserve University School of Medicine, Cleveland, OH 44106, USA; ^5^ Case Comprehensive Cancer Center, Case Western Reserve University School of Medicine, Cleveland, OH 44106, USA

**Keywords:** antigen amplification, tamoxifen, estrogen receptor antagonist, vaccine, cell-mediated immunotherapy

## Abstract

Immunotherapy has historically been successful in highly antigenic tumors but has shown limited therapeutic efficacy in non-antigenic tumors such as breast cancers. Our previous studies in autoimmunity have demonstrated that increased antigen load within a tissue enhances immune reactivity against it. We therefore hypothesized that enhancing expression of target proteins on breast tumors can increase efficacy of targeted immunotherapy. We hypothesized that antagonism of the estrogen receptor (ER) can increase expression of targets that are hormonally regulated and facilitate enhanced tumor recognition by targeted immunotherapy. We used a lactation protein α-Lactalbumin, a known immunotherapeutic target on breast tumors, as our model target antigen. Enhancement of target protein expression in human and murine breast tumors was tested *in vitro* and *in vivo* by ER antagonism using clinically established ER modulators, Tamoxifen and Fulvestrant. We show that antagonism of the ER can induce a 2–3 fold increase in expression of target proteins on tumors leaving the normal breast tissue unaffected. Tumor progression studies in 4T1 tumor-bearing mice show that efficacy of adoptively transferred cell based targeted immunotherapy was enhanced by target antigen amplification resulting in significantly higher tumor inhibition. However, in spite of increased target expression, anti-tumor efficacy of direct immunization was not enhanced probably due to other limiting factors involved in the immune priming process. Our study provides a novel combinatorial clinical strategy for enhancing efficacy of immunotherapy not only on breast tumors but potentially also for other hormonally driven tumors such as those of the prostate, testis and ovary.

## INTRODUCTION

Immunotherapy has historically been successful in highly antigenic tumors such as melanomas but has mostly encountered failure in non-antigenic tumors such as breast cancers [1, 2]. In spite of high efficacy in murine models, targeted immunotherapeutic strategies have mostly failed to translate this efficiency to the antigenically heterogeneous patient population encountered in the clinic. In fact, the limitation of recently FDA approved PD-1 and PD-L1 inhibitors to therapeutic efficacy to only 30% of patients [3] has brought to light the immense variation in efficacy of even such very powerful immunotherapeutic strategies. It is well recognized that efficacy of targeted antibodies such as Herceptin or PD-1 inhibitors is highly dependent on Her-2/PD-1expression levels on breast cancers. For cell-mediated immunotherapies, it has been shown that tumors with high mutation load are more antigenic and show greater responses to immunotherapeutic intervention [4]. The relation between high antigenicity and successful immunotherapy is thought to be due to higher infiltration of therapeutic immune cells into the tumor [5]. Furthermore, our own work in the experimental autoimmune encephalomyelitis murine model for multiple sclerosis has shown that increased antigen load within tissue leads to enhanced autoimmune reactivity targeted against it [6]. Although, increased antigenicity is considered to be a prerequisite for effective immunotherapy, there are no clinically viable strategies currently available to address this key issue and enhance target antigen expression on tumors. Most current immunotherapies circumvent the problem of inadequate target antigen expression by restricting patient population selection.

Augmentation of target antigen expression on tumors that have low or negative expression of the specific target can reduce the tumor heterogeneity encountered in the clinical setting particularly with regard to their antigenicity. In addition, it may widen the tumor population treatable by immunotherapy including otherwise unresponsive tumors. Increase in target expression on tumors would also increase antigenicity of individual tumors thereby enhancing the strength of immune responses against them. Low expression or downregulation of target antigen expression on tumors can be circumvented in two ways: (1) by adoptive transfer of large numbers of engineered T cells directed to a moderately expressed target antigen [7, 8] or (2) by increasing expression of the downregulated or poorly expressed target on the tumor. We hypothesize that expression of target proteins on tumors can be increased if their transcriptional signaling and control pathways can be identified and modulated. This would be especially feasible in hormone receptor driven tumors such as those of the breast, ovary, testis and prostate because of their amenability to low dose hormonal modulation without major side effects. It is well established that the growth and therapeutic response characteristics of breast tumors are strongly associated with the expression of estrogen (ER) and progesterone (PR) hormone receptors [9]. Since the breast, and majority of tumors derived from it are intricately controlled by hormone receptor signaling, we hypothesized that expression of a number of potential immunotherapeutic target proteins on breast tumors can be increased by modulation of hormone receptors controlling their transcription.

One such set of antigens, namely lactation proteins, are known to be under strict negative regulation via estrogen receptor signaling in order to facilitate timely post-partum lactation concurrent with a drop in estrogen levels [10, 11]. We selected a lactation protein, α-Lactalbumin as the model antigen to test our hypothesis. α-Lactalbumin is expressed at variable levels in 55–60% of all breast tumors [9, 12, 13] and immunization against it has been shown to provide significant inhibition of both spontaneous and transplanted breast tumors in murine models [14]. Oncomine database shows overexpression of α-Lactalbumin in 75% of Triple Negative Breast Cancer (TNBC) with much lower expression levels in ER+PR+ or ER+PR- breast tumors [15]. Based on these observations, we selected α-Lactalbumin as our model target on breast tumors to test our hypothesis that increase in target protein expression can be facilitated by modulation of hormone receptors on breast tumors which in turn can enhance efficacy of immunotherapy targeted against the tumor.

## RESULTS

### Antagonism of the estrogen receptor can increase gene transcription of hormonally regulated target antigens on breast tumors

**Figure 1:**
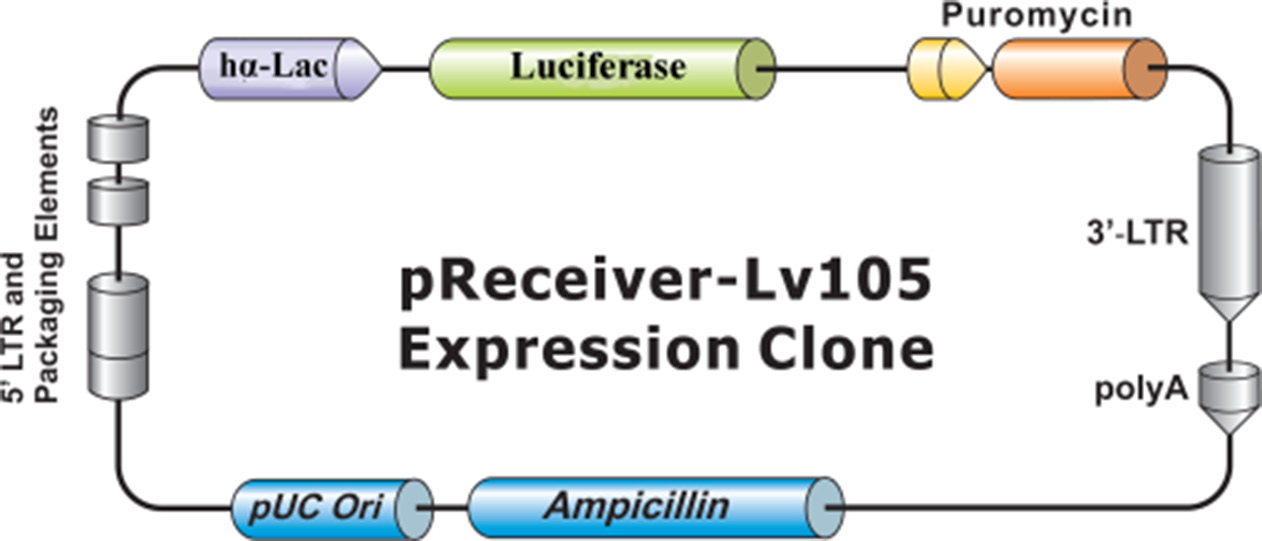
Map of pReceiver-Lv105 plasmid Map of the lentiviral expression vector encoding a Luciferase reporter gene under transcriptional control of the human α-Lactalbumin promoter (Genecopoeia Inc., Rockville, MD).

**Figure 2:**
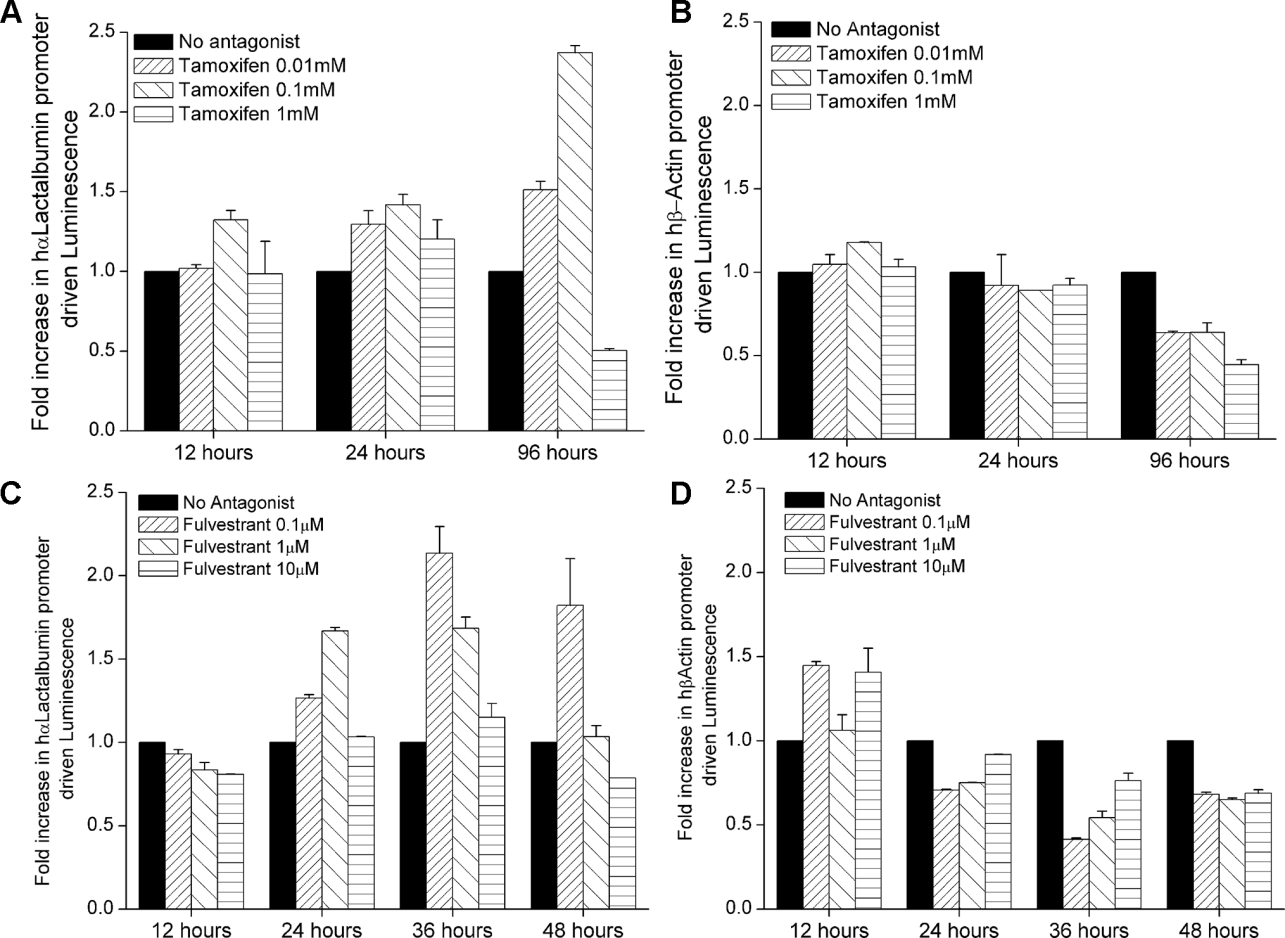
Estrogen receptor antagonism increases α-Lactalbumin promoter expression in breast tumors T47D-hLac-Luc (expressing luciferase under control of the human α-Lactalbumin promoter) or T47D-hBAct-Luc (expressing luciferase under control of the human β-Actin promoter) breast cancer cells were treated with Tamoxifen and Fulvestrant. Luminescence reading from treated cells were normalized to luminescence from untreated cells at each time point to obtain fold increase (*n* = 3 replicate experiments). Greater than 2 fold increase in α-Lactalbumin promoter activity was observed after treatment with Tamoxifen (**A**; *p* < 0.01 with 0.01 mM; *p* < 0.001 with 0.1 mM at 96 hours) and Fulvestrant (**C**, *p* < 0.01 with 0.1 μM at 36 hours; *p* < 0.1 at 48 hours). No increase in β-Actin promoter driven luminescence was observed after treatment with Tamoxifen (**B**) or Fulvestrant (**D**).

We sought to address our hypothesis that immunotherapeutic target antigens on hormonally regulated tumors can be upregulated or amplified in expression using hormone receptor modulation. In order to analyze changes in gene promoter activity upon ER antagonism, we engineered T47D cell lines to stably express Luciferase under transcriptional control of the α-Lactalbumin promoter (T47D-hLac-Luc; Figure 1) or the housekeeping β-Actin gene promoter (T47D-hBAct-Luc). We treated 1.5 × 10^5^ T47D-hLac-Luc or T47D-hBAct-Luc cells/well with different doses of a standard FDA approved selective estrogen receptor modulator Tamoxifen or a complete estrogen receptor antagonist Fulvestrant. Since Tamoxifen Citrate has a short half-life, it was renewed in media every 48 hours. Cells were assayed using the Bright Glo^™^ Luciferase assay for human α-Lactalbumin or human β-Actin promoter driven Luciferase activity post ER antagonism. Luminescence readings from ER antagonist treated cells, normalized to vehicle only treated cells revealed approximately 2–2.5 fold increase in expression of α-Lactalbumin promoter activity over endogenous expression after ER antagonism both by Tamoxifen (Figure 2A) and by Fulvestrant (Figure 2C). The increase in α-Lactalbumin promoter expression was dose-dependent for both drugs with maximum efficacy at 0.1 mM for Tamoxifen, 96 hours after beginning of treatment (*p* < 0.001) and also evident at a 1 log lower dose of 0.01 mM (*p* < 0.01). After Fulvestrant treatment, antigen upregulation was seen as early as 24 hours of treatment with maximum efficacy after 36 hours of treatment with a doses of 0.1μM Fulvestrant (*p* < 0.01). The effect continued till 48 hours after beginning of Fulvestrant treatment (*p* < 0.1). As expected, no substantial change in β-Actin promoter activity was observed after ER antagonism with Tamoxifen (Figure 2B) or Fulvestrant (Figure 2D). Observed decrease in β-Actin promoter-driven luminescence at higher doses and 96 hours after treatment may be due to the known cytostatic effect of Tamoxifen on hormone-dependent breast tumors. These *in vitro* studies provide proof for the concept that estrogen receptor antagonism can enhance transcription of hormonally regulated target antigens on breast tumors.

### Estrogen receptor modulation can increase expression of hormonally regulated target proteins on breast tumors

**Figure 3:**
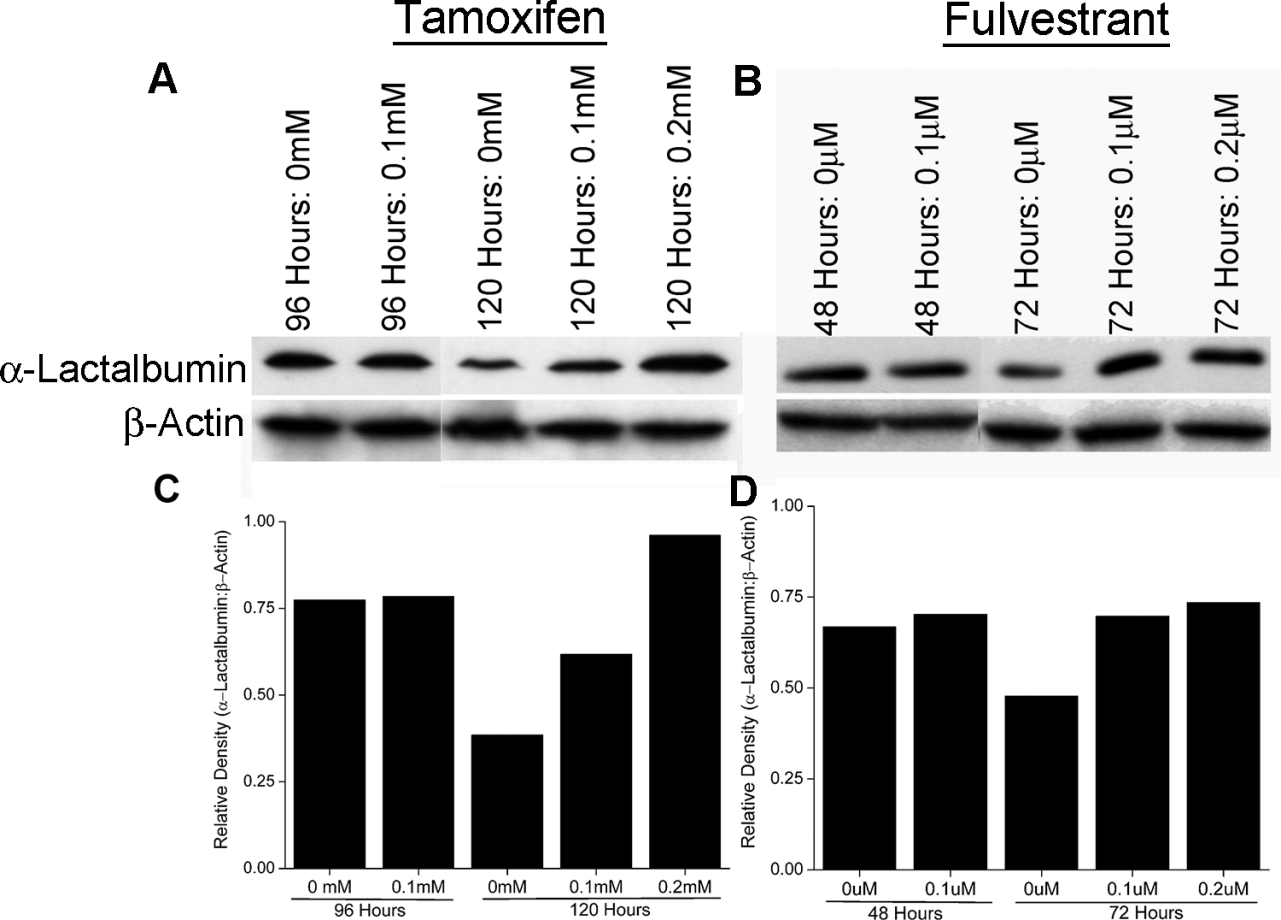
ER antagonism facilitates amplification of protein target expression on breast cancer cells T47D breast cancer cells were treated *in vitro* with Tamoxifen and Fulvestrant. At different time points of treatment, cells were lysed and analyzed for α-Lactalbumin expression by Western blotting. An at least 2 fold increase in α-Lactalbumin protein expression is seen in human breast cancer cells after 120 hours of Tamoxifen treatment (**A**, *Upper Panel*). A modest increase in α-Lactalbumin target protein expression is also seen at 72 hours after Fulvestrant treatment (**B**, *Upper Panel*). No change in expression of the housekeeping protein β-actin was observed (A, B, *Lower Panels*). Densitometry quantification of α-Lactalbumin specific Western blots post Tamoxifen (**C**) or Fulvestrant (**D**) treatment of T47D breast cancer cells.

To determine if increase in promoter activity facilitated by ER antagonism (shown above) translates to increased expression of the encoded protein, ER+PR+ T47D breast cancer cells were treated with Tamoxifen or Fulvestrant at 0.1 mM and 0.1 μm respectively (optimal doses as determined above). T47D breast cancer cells endogenously express the α-Lactalbumin target and were not transfected with any additional vector expressing α-Lactalbumin. Cell pellets were harvested at different time points after beginning of antagonist treatment, lysed and changes in endogenous α-Lactalbumin protein expression quantified. Western blot analysis reveals an increase in endogenous α-Lactalbumin protein expression 120 hours after Tamoxifen treatment (Figure 3A, *Top Panel*). In contrast, treatment of cells with the complete ER antagonist Fulvestrant, led to only a modest increase in α-Lactalbumin protein expression (Figure 3B, *Top Panel*. As expected, β-Actin protein expression remained unchanged after treatment with either antagonist (Figure 3A and 3B, *Bottom Panels*). Densitometric quantification of Western blots shows an at least 2.5-fold increase in α-Lactalbumin protein expression after 120 hours of Tamoxifen treatment (Figure 3C) and confirmed a modest increase after Fulvestrant treatment (Figure 3D). The observed 2.5-fold increase in α-Lactalbumin target protein expression, at 120 hours of Tamoxifen treatment is consistent with the observed increase in its promoter activity at 96 hours post treatment (Figure 2A). Results from these experiments prove that antagonism of the ER *in vitro* can facilitate an increase in target protein expression on breast tumors. Noticeably, cells not treated with antagonist show a decrease in target antigen expression during later stages of progression, a well-recognized strategy of immune escape by tumors (Figure 3A and 3B, *Top Panels*). In this context, it is important to note that antagonism of the ER could rescue endogenous target antigen expression at least to the original levels present in the tumor.

### Systemic administration of estrogen receptor antagonist, tamoxifen is effective in increasing expression of target antigens on breast tumors *in vivo*

**Figure 4:**
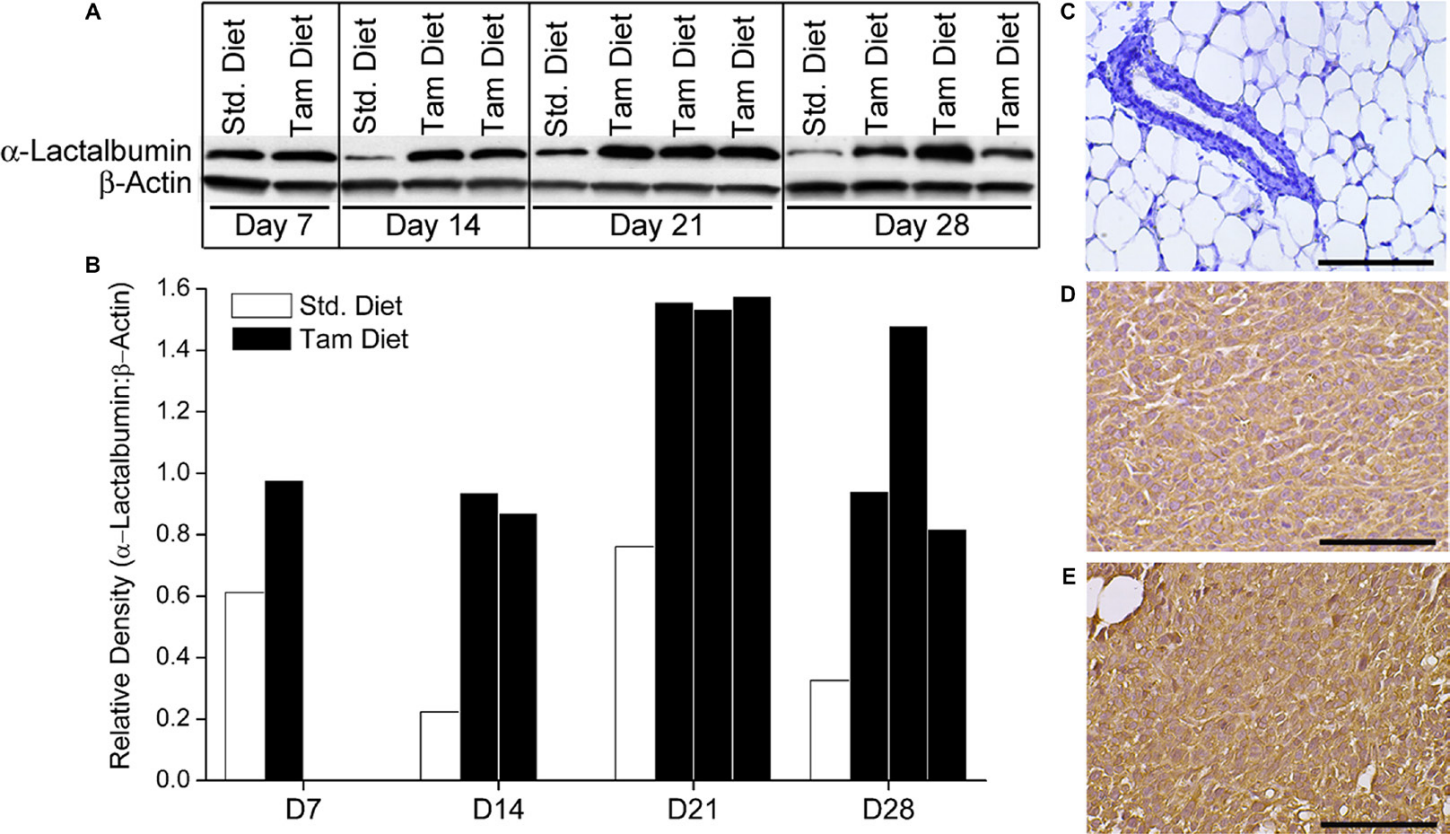
Systemic administration of the ER antagonist Tamoxifen can increase expression of immunological targets such as α-Lactalbumin on breast tumors *in vivo* Balb/cJ female mice on Standard or Tamoxifen containing diet were injected *s.c*. with 1 × 10^4^ 4T1 murine breast tumor cells. Mice were euthanized at day 7, 14, 21 and 28 post tumor inoculation and tumors analyzed for α-Lactalbumin protein expression. Significant increase in protein expression is observed in Tamoxifen treated 4T1 tumors compared to tumors from mice on standard diet, beginning at day 7 post tumor inoculation and continuing till end of the experiment at day 28 (**A**). Densitometry quantification of Western blots shows 4X amplification of α-Lactalbumin protein expression on day 14 of tumor growth (**B**). β-Actin was used as a loading and housekeeping protein control on the same blot. IHC shows substantially increased α-Lactalbumin staining (*brown color DAB*) in 4T1 tumors treated with Tamoxifen (**E**) compared to tumors from mice on standard diet (**D**). No α-Lactalbumin expression was evident in normal breast tissue (*blue hematoxylin counterstaining*) derived from Tamoxifen treated 4T1 tumor bearing mice (**C**). Bars depict 100 μm.

Balb/cJ female mice were maintained on Tamoxifen or Standard diet for the duration of the experiment. Ten days after beginning Tamoxifen diet, mice were transplanted with 1 × 10^4^ 4T1 murine breast tumors that are known to have moderate levels of endogenous α-Lactalbumin expression. Subsets of mice from each diet group (*n* = 3 per time point) were euthanized at days 7, 14, 21 and 28 after tumor injection. Tumors were removed, lysed and analyzed for expression of the target protein by Western blotting. 4T1 tumors from mice treated systemically with Tamoxifen showed a substantial increase in endogenous α-Lactalbumin protein expression compared to 4T1 tumors derived from mice on standard chow, beginning as early as two weeks after tumor transplant (Figure 4A, 4B, *Top Panels*. As expected, no change in expression of the housekeeping β-Actin protein was observed (Figure 4A, 4B, *Bottom Panels*. IHC staining with α-Lactalbumin-specific antibodies showed substantially increased protein expression in 4T1 tumors of mice treated with Tamoxifen diet (Figure 4E) compared to those from mice fed on standard diet (Figure 4D) with no changes in protein localization. It is noticeable that no alteration in α-Lactalbumin protein expression was evident in normal breast tissue derived from tumor-bearing mice receiving systemic Tamoxifen (Figure 4C). Results demonstrate that systemic *in vivo* delivery of Tamoxifen is effective in amplifying endogenously expressed levels of immunotherapeutic targets such as α-Lactalbumin selectively on hormone receptor positive breast tumors, with no deleterious bystander effect on protein expression in normal breast tissue. More importantly these results demonstrate the feasibility of amplifying naturally present target expression on tumors without using vector mediated overexpression experimental systems to modify the tumor.

### Amplification of target expression facilitated by ER antagonism could not enhance efficacy of active antigen-specific vaccination against breast tumors

**Figure 5:**
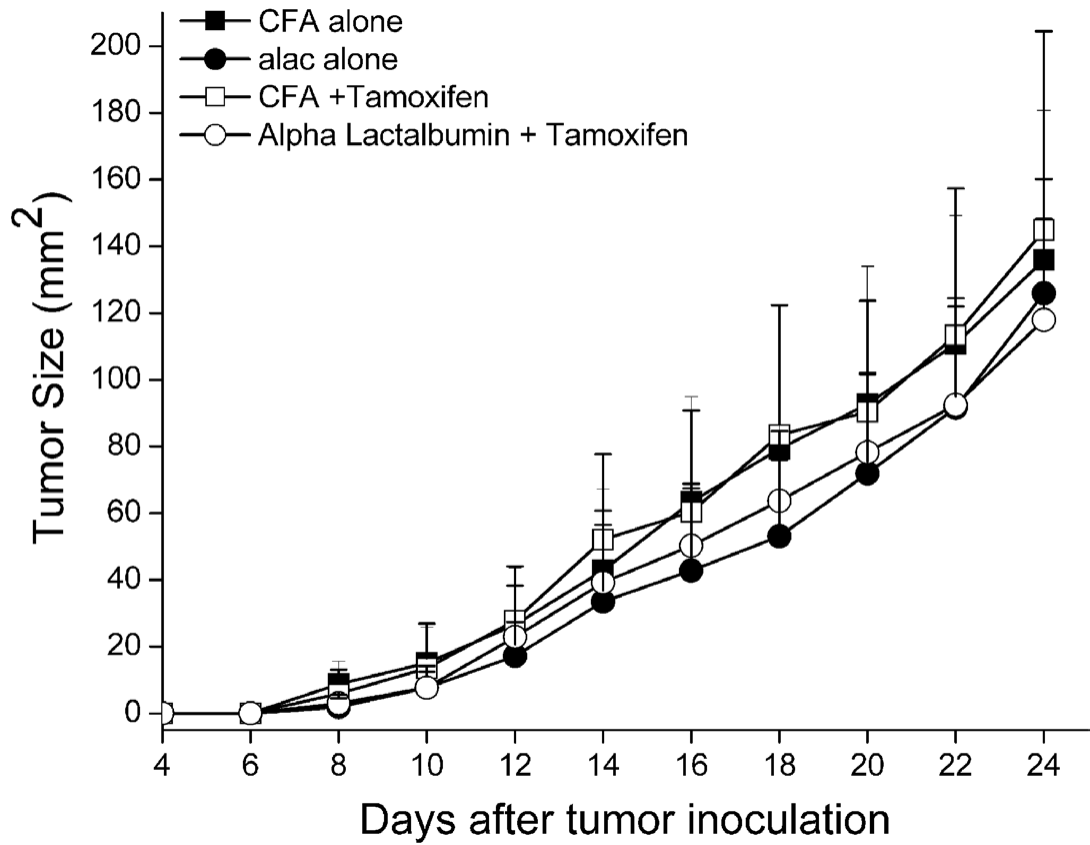
Increase in target antigen expression facilitated by ER antagonism does not enhance efficacy of direct antigen specific vaccination against breast tumors Balb/cJ female mice on Standard or Tamoxifen diet were transplanted with 4T1 breast tumors (*n* = 8). Five days post tumor inoculation, mice were immunized with α-Lactalbumin in CFA adjuvant or CFA alone. Tumors were measured every other day until they reached 17 mm size. Tumor area was calculated as length × breadth and plotted with time. Antigen amplification did not confer any increase in anti-tumor efficacy of active vaccination against α-Lactalbumin.

We hypothesized that amplification of target antigen on tumors would enhance the efficacy of antigen-specific, targeted vaccination against breast tumors expressing the enhanced protein target. To test our hypothesis, 5 week old Balb/cJ female mice were placed on standard or Tamoxifen diet and injected with 1 × 10^4^ 4T1 mammary cancer cells as described above. Five days post tumor inoculation, mice were immunized with either α-Lactalbumin in CFA or CFA adjuvant alone. Tumors were measured every other day until they reached 17mm size in either direction of measurement. No significant difference in therapeutic efficacy as a result of antigen-specific active vaccination alone or vaccination combined with Tamoxifen administration was observed (Figure 5). As expected, vaccination against α-Lactalbumin led to a modest therapeutic effect and slowing of tumor growth. However, increase in target antigen expression on breast tumors by Tamoxifen treatment afforded no added benefit to the therapeutic efficacy of direct vaccination.

### Amplification of target expression by ER antagonism enhances efficacy of targeted, cell mediated immunotherapy against breast tumors

**Figure 6:**
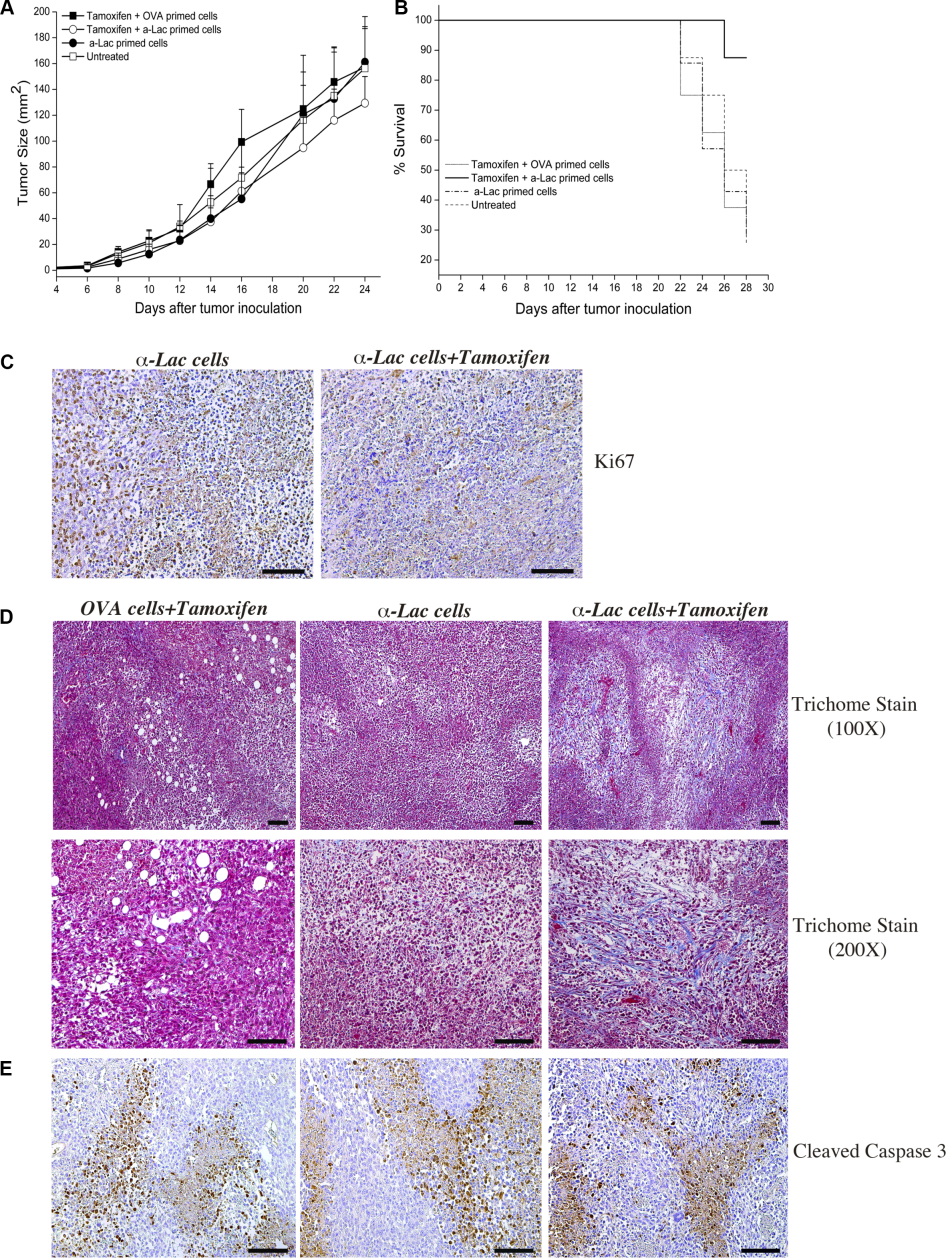
Amplification of target expression by ER antagonism enhances efficacy of antigen specific cell mediated immunotherapy against breast tumors Significant (*p* < 2 × 10^−6^) decrease in 4T1tumor progression was observed after treatment with Tamoxifen+α-Lactalbumin (α-Lac) primed lymphocytes (*open circles*) compared all other test groups (*n* = 8 each) (**A**). 4T1 tumors treated with α-Lactalbumin specific lymphocytes alone showed anti-tumor efficacy during initial tumor growth (*closed circles*), however only tumors treated with α-Lactalbumin primed lymphocytes+Tamoxifen continued to show enhanced therapeutic efficacy. Mice treated with Tamoxifen+α-Lac primed cells showed 87% survival at end of follow up at day 28 compared to 25% in controls (**B**; *p* < 0.04). Combination of α-Lac primed cell transfer with Tamoxifen diet resulted in significant reduction in cell division within 4T1 tumors as evident by Ki67 staining (**C**, *Right Panel*) compared to α-Lac specific cells alone (C, *Left Panel*). Tamoxifen diet+antigen specific cell transfer induced significant fibrosis (*blue Trichome stain*) in 4T1 tumors (**D**, *Right Panels*) compared to Tamoxifen+OVA primed cell transfer (D, *Center Panels*) or α-Lactalbumin primed cells alone (D, *Left Panels*). No significant differences in Cleaved Caspase 3 staining (*brown DAB stain*) were observed between treatment groups (**E**). Bars depict 100 μm.

Factors other than antigen expression, such as dendritic cell trafficking, antigen uptake and processing, adjuvants and depot effect etc., are critical for efficient T cell priming and success of the direct immunization process. We hypothesized that these factors could limit the benefit afforded by our proposed target amplification strategy during direct vaccination. We therefore tested anti-tumor therapeutic efficacy using adoptive transfer of antigen-specific lymphocytes in order to eliminate requirement of such factors. This allowed us to assess the impact of a singular factor (i.e., increase in target antigen expression) on targeted immunotherapy. Mice on Tamoxifen or standard diet were inoculated with 4T1 tumors and transferred *i.p*. with 20 × 10^6^ α-Lactalbumin or Ovalbumin (OVA) primed lymphocytes instead of direct subcutaneous vaccination. Follow-up of tumor progression over time revealed significant inhibition of tumor growth in mice on Tamoxifen diet transferred with α-Lactalbumin primed lymphocytes (Figure 6A). Mice receiving α-Lactalbumin primed cells but fed on standard diet showed tumor inhibition efficacy only during initial tumor growth, but the therapeutic effect was lost after day 16, probably concurrent with the expected downregulation of target by the tumor. Mice on Tamoxifen diet transferred with α-Lactalbumin primed lymphocytes, however, continued to show enhanced therapeutic efficacy compared to untreated or Tamoxifen treated mice transferred with non-specific antigen (Ovalbumin) primed lymphocytes. More importantly, Tamoxifen fed mice receiving α-Lactalbumin primed lymphocytes showed approximately 90% survival at day 28 compared to 25% survival in the group receiving α-Lactalbumin primed lymphocytes alone or on standard diet (Figure 6B). Experiments had to be terminated at day 28 post tumor inoculation because tumors in control groups had reached the 17mm endpoint as per IACUC regulations. Tumors harvested from test and control mice at the same time point were tested by IHC for evaluation of tumor inhibition as well as immune mediators involved in the process.

Combination of α-Lactalbumin primed cell transfer with Tamoxifen diet resulted in significant reduction in 4T1 tumor cell division as evidenced by Ki67 staining (Figure 6C, *Right Panel*) compared to tumors treated with α-Lactalbumin specific cells alone (Figure 6C, *Left Panel*). Tumors treated with Tamoxifen diet plus α-Lactalbumin specific cell transfer showed significant increase in fibrosis and tumor necrosis (blue Trichome staining, red cytoplasm, black nuclei; Figure 6D, *Right Panels*) compared to tumors treated with α-Lactalbumin alone (Figure 6D, *Center Panels*) or non-specific OVA primed cells plus Tamoxifen (Figure 6D, *Right Panel*). No differences in apoptosis as evidenced by Cleaved Caspase 3 staining were observed with any of the treatments (Figure 6E).

### Amplification of target expression by ER antagonism enhances infiltration of B220+ positive cell infiltrate within tumors treated with antigen specific lymphocyte transfer

**Figure 7:**
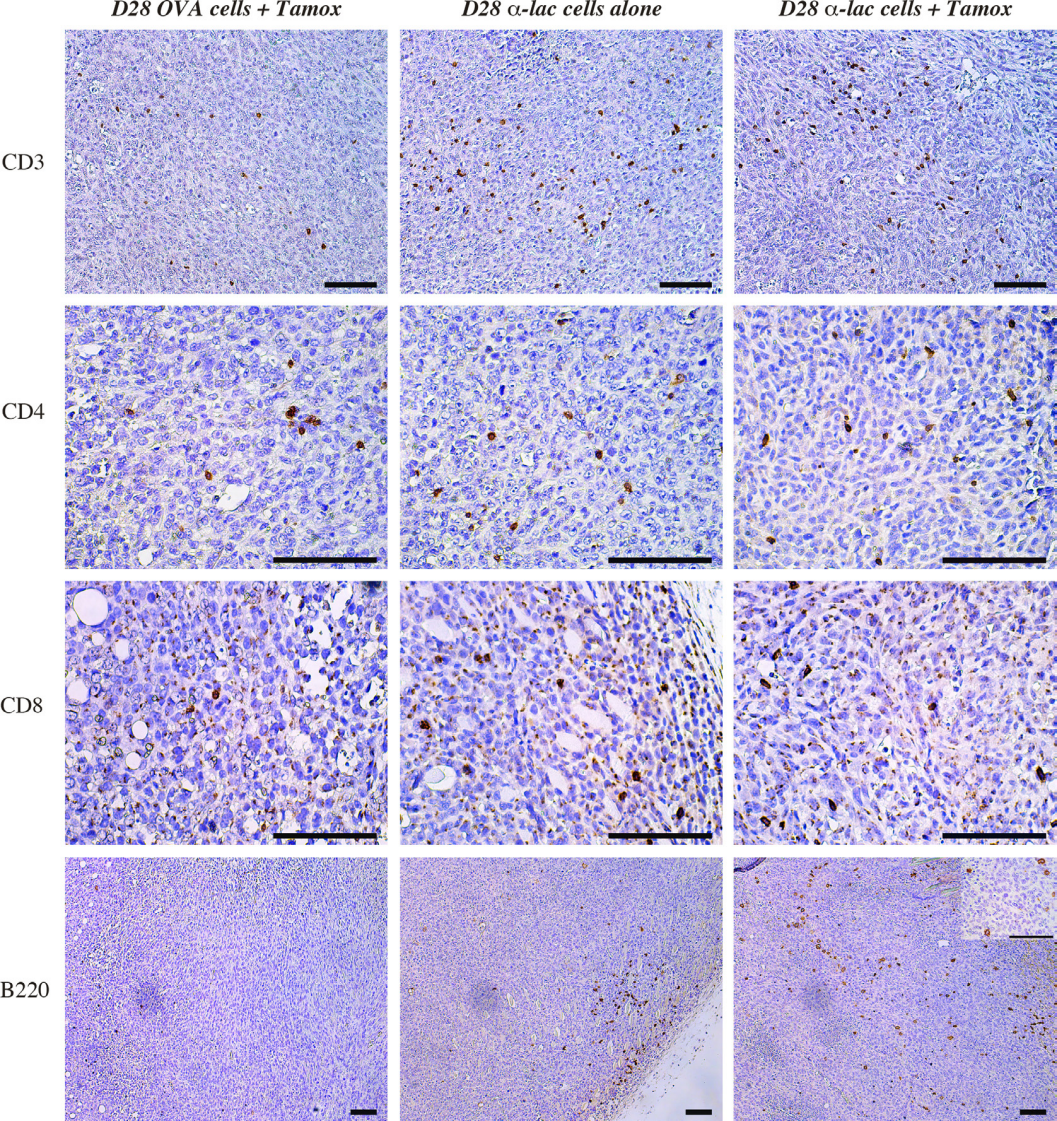
Amplification of target expression by ER antagonism enhances infiltration of B220+ positive cell infiltrate within tumors Immunohistochemistry shows increase in CD3+ (*Top Panel*), CD4+ (*Second Panel*) and CD8+ (*Third Panel*) cells in tumors treated with α-Lactalbumin primed lymphocytes+Tamoxifen (*Right Vertical Panel*) and α-Lactalbumin specific lymphocytes alone (*Center Vertical Panel*) compared to non-specific (OVA) antigen primed cells (*Left Vertical Panel*). Although no increase was observed in CD3+, CD4+ and CD8+ cell infiltration in α-Lactalbumin primed lymphocytes+Tamoxifen treated tumors compared to those treated with α-Lactalbumin primed lymphocytes alone, significant increase in B220+ cell infiltration was observed between the two groups (*Bottom Panel*). Insert shows 4× magnification of B220 stain showing plasmacytoid dendritic cell like morphology. Bar depicts 100 μm on all panels.

Previous work on α-Lactalbumin vaccination has shown amplification of and involvement of both CD4+ and CD8+ cells in the anti-tumor immune response post α-Lactalbumin targeted vaccination (14). Investigation into immune mediators of the observed increase in tumor fibrosis and cell death in Tamoxifen+ α-Lactalbumin, revealed a significant increase in CD3+ T cells, CD4+ and CD8+ infiltrates in tumors treated with α-Lactalbumin specific cell transfer compared to those treated with non-specific antigen (OVA) primed lymphocytes (Figure 7, *Top three panels*). However, CD3 infiltrates could not account for enhanced tumor inhibition observed in mice receiving Tamoxifen in addition to the α-Lactalbumin primed cells compared to those receiving α-Lactalbumin primed cells alone with no antigen amplification. Immunohistochemical staining for B220+ cells showed a significant increase in B220+ cell infiltrates in tumors treated with Tamoxifen+ α-Lactalbumin primed cells > α-Lactalbumin primed cells alone > non-specific antigen primed cells (Figure 7, *Bottom Panel*). Examination at higher magnification of infiltrating B220 cells in tumors treated with Tamoxifen and target antigen specific cells shows large cells with dendrites and a plasmacytoid Dendritic Cell (pDC) like morphology (Figure 7, *Bottom Panel insert*). We further conducted cytokine analyses of the tumor microenvironment (*Data not shown*). No differences were seen in cytokine levels between α-Lactalbumin specific cell treated tumors with or without Tamoxifen treatment. We believe that this could be due to the enhanced cell death and fibrosis in antigen amplified tumors at the late stage of tumor growth in these experiments indicating enhanced cytotoxic activity of infiltrating T cells in the tumor bed. These studies demonstrate that amplification of target antigen expression on breast tumors is effective in enhancing expression of target antigen in tumors, possibly leading to enhanced recruitment of antigen presenting cells into the tumor microenvironment that facilitates enhanced cytotoxic T cell activity and increased efficacy of antigen-specific immunotherapy.

## DISCUSSION

Our study provides a novel strategy for enhancing efficacy of tumor immunotherapy by amplification of the target on tumors. We show for the first time that expression of certain target antigens on hormone receptor positive breast tumors can be amplified by modulation of their hormone receptor regulated transcriptional processes. We show that such target amplification can be easily facilitated by antagonism of the estrogen receptor with clinically approved and established estrogen receptor modulators such as Tamoxifen. Our results suggest that combination immunotherapies for breast cancer incorporating Tamoxifen could not only enhance efficacy of targeted immunotherapy against ER+PR+ breast tumors but also expand the repertoire of “effectively targetable tumors” by immunotherapy to include tumors with otherwise negative or low target antigen expression. Our data provide experimental proof that level of antigen expression, especially later during tumor progression, is an important parameter in determining efficacy of antigen targeted anti-tumor immunotherapy. We provide a possible strategy to circumvent the down regulation of target antigen expression as a mode of tumor escape by simply modulating the transcriptional control of the target antigen by established and currently available drugs.

Our proposed strategy is especially significant in the context of breast tumors since estrogen receptors are known to be overexpressed in approximately 75% of breast cancers. Signaling through the estrogen receptor controls and initiates gene expression by interactions with basal transcription factors and is strongly associated with tumorigenesis [16]. The progesterone receptor also functions as a nuclear transcription factor controlling a large number of genes; however, it is controlled and induced by ER signaling in normal reproductive tissue as well as in breast tumors [17, 18]. In the normal breast, relatively few cells express ER and PR with an inverse relationship between receptor expression and proliferation. Increased expression of the hormone receptors and a loss of this inverse relationship between receptor expression and cell division are thought to be at the inception of transformative processes [19]. Estrogen receptor antagonists inhibit these conformational changes required for signaling through the ER [20]. Therefore, modulation of this transcriptional control of protein targets on tumors via FDA-approved ER/PR antagonists can be a viable and effective strategy to increase their availability for targeted immunotherapy. Even though substantial gene transcription in breast tumors is controlled by the PR, we focused on antagonizing only the estrogen receptor since PR expression is heavily dependent on ER expression where only 1–2% of breast tumors are known to be ER-PR+ [21, 22]. We show here that efficacy of immunotherapy against breast tumors can be enhanced by combination therapy with already FDA approved drugs like Tamoxifen and Fulvestrant; known estrogen receptor modulators with very tolerable side effects, thereby extending the potential clinical applications of these drugs.

Tamoxifen is a selective estrogen receptor modulator (SERM) approved for adjuvant therapy of breast cancer and has been shown to significantly increase recurrence free survival [23]. In the breast, Tamoxifen acts primarily as an antagonist, whereas in bone, liver, and the uterus, it acts primarily as an estrogen agonist [24, 25, 26]. In contrast to Tamoxifen, which can have both antagonist and agonist effects, Fulvestrant is a complete ER downregulator that competitively binds to the ER to prevent estrogen mediated signaling and accelerates proteosomal degradation of the receptor [27]. Fulvestrant is approved for second-line chemotherapy for postmenopausal women with metastatic breast cancer and relapse or disease progression following anti-estrogen therapy [28]. In the current study, even though Fulvestrant treatment facilitated an increase in antigen expression within 24–36 hours of treatment, it translated into only a modest increase in protein expression. We believe that this could be due to higher cell toxicity associated with complete ER antagonism. Tamoxifen treatment in contrast showed a delayed effect on antigen upregulation owing to the 4 day lag period required for reaching steady state levels in most biological systems. However, treatment with Tamoxifen resulted in at least a doubling of protein expression at around 5 days of treatment, coinciding with the peak increase in its promoter activity approximately 4 days after beginning of treatment (Figures 2A and 3A, 3C).

Down regulation of target antigens is a well-recognized mode of tumor escape and a major reason for failure of targeted therapies especially when administered during later stages of tumor progression. In fact, we observed a decrease in endogenous α-Lactalbumin expression in both T47D human tumors grown *in vitro* and 4T1 murine tumors grown *in vivo* at later stages of growth (Figures 3 and 4). Cell-mediated immunotherapy with no assisted antigen amplification was effective during initial stages of tumor progression, beyond which, the therapeutic effect was lost either due to lack of antigen specific cell survival or as shown in our experiments, downregulation of target antigen by a growing and immune evasive tumor. We show that ER antagonism was effective in increasing levels of down-regulated target protein to at least initial expression levels or more on the tumor. With constant transcriptional modulation of antigen expression via Tamoxifen-mediated ER antagonism, we could achieve long lasting anti-tumor efficacy that translated into significantly increased survival. More importantly we demonstrate the ability to increase endogenous target antigen expression on tumors without using manipulated/ antigen overexpression tumor systems. These data provide proof of concept for the possibility of circumventing tumor immune escape mechanisms clinically using simple strategies such as modulation of hormone receptor signaling and transcription of immunotherapeutic targets.

We demonstrate that a singular increase in antigen expression on tumors can result in enhanced efficacy of targeted cell-based tumor immunotherapy. However, we did not observe any improvement in therapeutic efficacy of direct subcutaneous vaccination in spite of a doubling in target protein expression. We attribute this lack increased efficacy after direct vaccination to numerous rate-limiting immunological parameters involved in efficient T cell priming and activation. Direct vaccination with subcutaneous injection of antigen in emulsion with adjuvants, is highly dependent on availability and efficient trafficking of antigen-presenting dendritic cells to the site of injection and draining lymph nodes. Furthermore, subsequent antigen processing and presentation in the context of MHC-Class II molecules and cross presentation by MHC-Class I expressing tumor cells determines the efficacy of T cell priming. We believe that the effect of a sole increase in target antigen expression would be masked by limitations posed by above mentioned immunological factors in spite of its importance in anti-tumor immunity. In fact we show that the most significant difference between immune mediators in antigen targeted cell transfer +Tamoxifen treated tumors vs tumors with antigen targeted cell transfer alone was seen in infiltration of plasmacytoid dendritic cells within the antigen amplified tumor. Composition of the α-Lactalbumin vaccine mediated immune response has been previously characterized extensively and has been shown to be mediated by both CD4+ and CD8+ cells as was also seen in the current study with increased infiltration of these cells in α-Lactalbumin treated tumors compared to OVA treated tumors. Interestingly no differences were seen in numbers of CD3+, CD4+ or CD8+ cells infiltrating the antigen amplified vs non amplified α-Lactalbumin treated tumors (Figure 7). However, their enhanced cytotoxic activity in Tamoxifen treated tumors is evident in the observed increase in tumor cell death and fibrosis (Figure 6). We also did not observe significant differences in pro-inflammatory cytokine levels in the above mentioned tumors (Data not shown). We speculate this to be due to assay of late stage tumors in advanced stage of fibrosis, where the peak of immunological action has ceased at that snapshot in time. Tumor taken at earlier time points would be ideal material for future mechanistic studies.

Lactation proteins have been recently recognized as viable target antigens for breast tumor immunotherapy since they are expressed in the normal breast only during lactation but expressed in a substantial number of breast tumors. Lactation proteins as tumor targets are especially amenable to our proposed strategy of antigen amplification via ER antagonism since their production is known to be highly regulated by the estrogen and progesterone receptor signaling. Expression of lactation proteins is known to increase concurrently with a drop in estrogen and progesterone signaling in order to facilitate the lactation spurt post parturition. We therefore selected a lactation protein, α-Lactalbumin as our model antigen to test our hypothesis. Although we have used α-Lactalbumin vaccination as merely a model system in the current study, vaccination against α-Lactalbumin is currently under investigation for prevention of TNBC which form about 15–30% of all breast cancers. Our proposed strategy for enhancing antigen expression could extend the coverage provided by α-Lactalbumin vaccination, possibly to the remaining 75% patients with hormone receptor positive breast cancers.

Overall our study addresses a key and current issue in the immunotherapy field, i.e., enhancement of current efficacy of targeted immunotherapy and expansion of its success rate. We provide a novel, clinically applicable strategy that exploits overexpression of hormone receptors on tumors for enhancing efficacy of targeted immunotherapy. We provide for the first time experimental evidence for the stand-alone importance of antigen expression in determining efficacy of immunotherapy. In summary, our study shows a way for making targets on tumors larger and more frequent. Our proposed strategy has clinical and translational significance for tumor immunotherapy and is extendable to other hormonally driven cancers such as those of the prostate, ovary and testis.

## MATERIALS AND METHODS

### Cell lines and antagonists

Human breast carcinoma line T47D (ATCC^®^ HTB 133^™^) and mouse 4T1 (ATCC^®^ CRL2539^™^) and EMT6 (ATCC^®^ CRL2755^™^) mammary cancer lines were purchased from ATCC (Manassas, VA). All cell lines were authenticated by ATCC through Short Tandem Repeat (STR) analysis prior to purchase. All cell lines were used under 20 passages. Tamoxifen Citrate (Sigma Aldrich, St. Louis, MO) was reconstituted in DMSO and used in culture at concentrations ranging from 0.01 mM to1 mM. Tamoxifen Citrate was administered *in vivo* murine studies via diet pellets containing 400 mg Tamoxifen Citrate per kg of diet (Harlan Laboratories, Indianapolis, IN). Fulvestrant was commercially procured (Sigma Aldrich), reconstituted in DMSO and used in cultures at concentrations ranging from 0.1 μM to10 μM.

### Generation of lentivirus modified luciferase reporter human breast cancer cells

Lentiviral particles harboring the pReceiver-Lv105 plasmid (Figure 1) were custom synthesized (GeneCopoeia Inc., Rockville, MD). Two separate plasmids were constructed to express the Luciferase enzyme under the transcriptional control of the human α-Lactalbumin promoter (Lentivirus hLac-Luc) or the human β-Actin promoter (Lentivirus hBAct-Luc). 5 × 10^6^ T47D cells were co-incubated with 5 × 10^6^ plaque forming units of either Lentivirus hLac-Luc or Lentivirus hBact-Luc for 24 hours to generate stable human breast cancer reporter cells expressing Luciferase regulated by the human α-Lactalbumin promoter (T47D-hLac-Luc) or human β-Actin promoter (T47D-hBAct-Luc). Stable transfectants were selected with 2.5 μg/ml puromycin and used for further studies. Presence of vector and stable modification was confirmed by Bright Glo™ Luciferase assay (Promega, Madison, WI).

### *In vitro* studies on upregulation of antigen-specific promoter activity

T47D-hLac-Luc or T47D-hBAct-Luc, ER+PR+ breast cancer cells were seeded at 1.5 × 10^5^ cells/well and treated with different doses of Tamoxifen Citrate or Fulvestrant or Dimethyl Sulphoxide (DMSO) vehicle only. Tamoxifen Citrate was renewed in the cultures every 48 hours. At 12, 24, 36, 48, 96 hour time points after beginning Tamoxifen/Fulvestrant treatment, cells were assayed using the Bright Glo™ Luciferase assay (Promega) containing Luciferin as substrate for α-Lactalbumin or β-Actin promoter driven Luciferase expression. Luminescence readings from antagonist treated cells were normalized to readings from vehicle treated cells at each time point and plotted as fold increase in promoter driven luminescence/Luciferase expression. Luminescence measured from T47D-hLac-Luc cells was compared to luminescence from equal numbers of similarly treated un-modified T47D cells or T47D cells with β-Actin promoter driven Luciferase expression (T47D-hBAct-Luc cells).

### *In vitro* studies on upregulation of target protein expression

T47D-hLac-Luc or T47D-hBAct-Luc, ER+PR+ breast cancer cells were treated *in vitro* with Tamoxifen Citrate or Fulvestrant or DMSO vehicle control as described above. 8 hours before each harvest time point specified above, cultures were treated with 1X protein transport inhibitor cocktail containing Brefeldin A and Monensin (eBioscience, San Diego, CA) to retain endogenously expressed α-Lactalbumin in the cell for assaying. 2 × 10^6^ antagonist treated T47D-hLac-Luc or T47D-hBAct-Luc cells were lysed in Mammalian Protein Extraction Reagent (M-PER; Thermo Fisher, Waltham, MA) and analyzed by Western blot analysis using α-Lactalbumin and β-Actin specific antibodies as described below.

### Mice and antagonist treatment

5–6 weeks old Balb/cJ female mice were commercially purchased (Jackson Laboratory, Bar Harbor, ME). All mice were housed in the Biologic Resources Unit of the Lerner Research Institute, Cleveland Clinic and treated as per protocols approved by the Institutional Animal Care and Use Committee (IACUC) of the Cleveland Clinic. Mice had unlimited access to food and water at all times. Mice treated with Tamoxifen were fed with specially formulated diet pellets containing Tamoxifen Citrate beginning at least 10 days prior to any tumor inoculations or vaccinations in order to allow animals to adjust to the food as well as attain constant systemic levels of Tamoxifen.

### Tumor inoculation and vaccination

1 × 10^4^ 4T1 mouse breast tumors were inoculated subcutaneously in 100 μl volume of PBS on the left side of the back. Mice were immunized with either Ovalbumin or α-Lactalbumin or Complete Freund's Adjuvant (CFA) alone as per design of the experiment. Ovalbumin was commercially purchased (Sigma Aldrich, St Louis, MO). Recombinant mouse α-Lactalbumin was purified as described previously (8). Recombinant purified protein was subject to reverse phase high performance liquid chromatography (HPLC) to yield endotoxin-free protein. Purified α-Lactalbumin or Ovalbumin were dissolved in water and mixed with CFA in a 1:1 water and oil emulsion using a syringe and stop-cock. Mice were immunized by bilateral subcutaneous injections of emulsion in the abdominal flank to deliver a total of 100 μg protein per mouse.

### *In vivo* antigen upregulation studies

Five-six week old Balb/cJ female mice were placed on Tamoxifen containing diet or normal chow at day 0 (D0). Ten days later, mice on either Tamoxifen diet or normal chow were inoculated with 1 × 10^4^ 4T1 mouse breast tumor cells subcutaneously as described above. Unmanipulated 4T1 tumor cells that express α-Lactalbumin endogenously were used for all experiments. Subsets of 4T1tumor bearing mice on either diet were euthanized at day 7, day 14, day 21 and day 28 post tumor inoculation. Tumors and normal breast tissue from mice at each time point were harvested and either fixed in formalin for immunohistochemistry or frozen for Western blot analysis.

### Quantification of target antigen upregulation

Endogenous α-Lactalbumin target antigen expression on 4T1 breast tumors post treatment with Tamoxifen was analyzed by Immunohistochemistry and further quantified by Western blot analysis using antigen-specific antibodies.

### Immunohistochemistry

5μm sections of Formalin fixed paraffin embedded (FFPE) 4T1 murine breast tumors or normal murine breast tissue were deparaffinized and heat mediated antigen retrieval performed at pH 6.1 in citrate buffer (Dako, Via Real Carpinteria, CA). Sections were treated with 5% Triton X-100, blocked in 3% normal goat serum for 1hr at room temperature and incubated at 4°C, overnight with polyclonal rabbit anti-mouse α-Lactalbumin antibody (Sigma Aldrich, St. Louis, MO, Catalog #HPA029856) followed by a goat anti-rabbit HRP labeled secondary antibody for 30 minutes at room temperature (Vector Labs, Burlingame, CA). Antibody binding was detected by incubation in Diaminobenzidine substrate (DAB; Vector labs) and sections counterstained with Harris Hematoxylin (Electron Microscopy Sciences, Hatfield, PA). Ki67 staining was performed with a rabbit anti-goat primary antibody (AbCam, Cambridge, MA, Catalog #ab15580). Cleaved Caspase 3 was detected by a commercially available monoclonal antibody (Cell signaling technology, Danvers, MA, Catalog #96645). Trichome stains (Masson) were performed according to the manufacturer's protocol (Sigma Aldrich). Anti CD3 (Bio Rad #MCA1477), CD4 (eBioscience #14-9766-80), CD8 (eBioscience #14-0808-80) and B220 (BD Pharmingen #550286) antibodies for immunological analyses were commercially obtained. All images were acquired on a Leica DM2000 LED using a DFC450C camera in the bright field setting at 100×, 200× and 400× magnifications. All images have a magnification scale bar that represents 100 μm.

### Western blot analysis

Frozen tumor and breast tissue were weighed, mechanically dissociated and lysed in M-PER solution followed by protein quantification using standard Bradford assay. 40 μg protein was electrophoresed on a 15% SDS-polyacrylamide gel (Bio-Rad Hercules, CA), transferred to a PVDF membrane (Millipore, Billerica, MA), blocked in 3% Bovine Serum Albumin (Sigma Aldrich, St. Louis, MO, Catalog #A7030) overnight and probed with rabbit anti-mouse primary antibodies against α-Lactalbumin (U.S. Biologicals, Salem, MA, Catalog #037664). Mouse anti-human β-Actin primary antibody (Proteintech, Rosemont, IL, Catalog #60008-1) was probed on the same blot as loading and protein expression control. Primary antibody binding was detected using HRP conjugated goat anti-rabbit (Santa Cruz Biotechnologies, Dallas, TX) or anti-mouse secondary antibodies (Promega) followed by detection of chemiluminescence on X-Ray films (Denville Scientific, Holliston, MA). Intensity of specific protein bands was quantified using the Image Studio software (LI-COR, Lincoln, Nebraska) and normalized to intensity of β-Actin control bands.

### T cell adoptive transfer

Five-six week old Balb/cJ female mice were immunized with 100 μg of α-Lactalbumin or Ovalbumin in CFA. Ten days post immunization primed lymph nodes were removed and dissociated into a single cell suspension. Ovalbumin or α-Lactalbumin primed lymphocytes were cultured at 5 × 10^6^ cells/well in 2 ml total culture volume and restimulated with 25 μg/ml of Ovalbumin or α-Lactalbumin respectively. 72 hours post restimulation, cells were harvested and 20 × 10^6^ Ovalbumin or α-Lactalbumin primed lymphocytes injected into 4T1 tumor bearing mice via intraperitoneal (*i.p*.) injections. All activated lymphocytes were transferred into tumor bearing mice at day 5 post tumor inoculation.

### Evaluation of breast tumor growth in mice

Mice subcutaneously inoculated with 4T1 tumors were immunized with α-Lactalbumin in CFA or with CFA alone or transferred with α-Lactalbumin/Ovalbumin primed lymphocytes. Tumors were measured using digital Vernier caliper every other day. Two measurements along the largest dimensions were taken perpendicular to each other to obtain tumor area expressed in mm^2^. Mice were monitored until tumors reached 17 mm in any direction at which time all mice were humanely euthanized as per recommendations of the IACUC of the Cleveland Clinic. Post euthanasia tumors were dissected and evaluated by IHC for indicators of growth inhibition such as Ki67, Cleaved Caspase 3 staining, T cell infiltration and fibrosis.

### Statistical analysis

Statistical analysis was performed using Origin 6.0 software (OriginLabs Corporation, Northampton, MA). Two-tailed students *t*-test was performed for determining significance of increase in promoter activity. Two tailed paired *t*-test was performed for significance determination on survival curves. Tumor growth curves were compared and significance established by using a one way ANOVA analysis. Results were considered statistically significant at a *P* value of < *0.05*.

## Abbreviations

ER/PR: Estrogen receptor/Progesterone Receptor
TNBC: Triple Negative Breast Cancer
α-Lac: Alpha Lactalbumin
β-Act: Beta Actin
OVA: Ovalbumin
Luc: Luciferase
CFA: Complete Freund's Adjuvant
HRP: Horse Radish Peroxidase
